# Exploring depression in Parkinson’s disease: an Italian Delphi Consensus on phenomenology, diagnosis, and management

**DOI:** 10.1007/s10072-023-06740-w

**Published:** 2023-04-27

**Authors:** Fabrizio Stocchi, Angelo Antonini, Paolo Barone, Giuseppe Bellelli, Andrea Fagiolini, Luigi Ferini Strambi, Sandro Sorbi, Alessandro Padovani

**Affiliations:** 1 University San Raffaele Rome and IRCCS San Raffaele, Rome, Italy; 2 Parkinson and Movement Disorders Unit, Center for Neurodegenerative Diseases (CENSE), Department of Neuroscience, University of Padua, Padua, Italy; 3 Department of Medicine and Surgery, Neuroscience Section, University of Salerno, Salerno, Italy; 4 School of Medicine and Surgery, University of Milano-Bicocca, 20126 Milan, Italy; 5 Acute Geriatric Unit, IRCCS San Gerardo, 20900 Monza, Italy; 6 Department of Molecular and Developmental Medicine, University of Siena, School of Medicine, Siena, Italy; 7 Department of Clinical Neurosciences, IRCCS San Raffaele Scientific Institute, Neurology-Sleep Disorders Centre, Milan, Italy; 8 Vita-Salute” San Raffaele University, Milan, Italy; 9 Department of Neurosciences, Psychology, Drug Research and Child Health, University of Florence, Florence, Italy; 10 IRCCS Fondazione Don Carlo Gnocchi, Florence, Italy; 11 Department of Clinical and Experimental Sciences, Neurology Unit, University of Brescia, Brescia, Italy

**Keywords:** Parkinson’s disease, Depression, Delphi Consensus, SSRI, Multimodal antidepressants

## Abstract

**Background:**

Depression is a prodromic and a frequent non-motor symptom of Parkinson’s disease, associated to reduced quality of life and poor outcomes. The diagnosis of depression in parkinsonian patients represents a challenge due to the overlapping of symptoms typical of the two conditions.

**Methods:**

A Delphi panel survey was performed to reach a consensus amongst different Italian specialists on four main topics: the neuropathological correlates of depression, main clinical aspects, diagnosis, and management of depression in Parkinson’s disease.

**Results and conclusion:**

Experts have recognized that depression is an established risk factor of PD and that its anatomic substrate is related to the neuropathological abnormalities typical of the disease. Multimodal and SSRI antidepressant have been confirmed as a valid therapeutic option in the treatment of depression in PD. Tolerability, safety profile, and potential efficacy on broad spectrum of symptoms of depression including cognitive symptoms and anhedonia should be considered when selecting an antidepressant and the choice should be tailored on the patients’ characteristics.

## Introduction 

Depression is a frequently encountered psychiatric disorder in Parkinson’s disease (PD), affecting approximately 40–50% of patients and assuming the characteristics of major depression in 17% of cases [1]. The presence of depression has in fact been associated with greater disability, faster cognitive decline, greater mortality, and a heavier burden on families and caregivers [2]. Depression can also precede the onset of motor symptoms, and it is now included as a risk factor in the diagnostic criteria for prodromal PD published in 2019 [3]. Indeed, the presence of depression as well as anxiety disorders has been associated to twofold increased risk of developing PD later in life [4].

The mechanisms underlying depression in PD are still not fully understood but autopsy and functional anatomy studies have demonstrated the presence of alterations in the limbic system and in the noradrenergic and serotonergic nuclei of the brainstem [5, 6].

This dual nature accounts for the partial resistance of depression to treatments based solely on dopaminergic replacement therapy and the partial ineffectiveness of common antidepressants which do not target alteration of the dopaminergic system [7].

The diagnosis of depression in the context of PD has always represented a challenge due to the overlapping between symptoms of depression and symptoms typical of the disease. The aim of this Delphi study is to reach a shared point of view amongst different Italian neurologists on the diagnosis and management of depression in PD based on Delphi Consensus. Four settings are explored: depression and neurodegenerative disease, clinical features of depression in AD, diagnostic criteria of depression in AD, and treatment of depression in AD.

## Methods

The Delphi method is a survey technique that uses responses to a standardized questionnaire developed by a panel of experts to facilitate the convergence of opinions or the achievement of a common opinion in areas where scientific evidence is scarce or needed [8]. The Delphi method involves the repeated administration of questionnaires, where each statement can be evaluated through a 5-point Likert scale, with a score from 1 to 5 (1, extremely disagree; 2, disagree; 3, agree; 4, mostly agree; and 5, extremely agree). Results are expressed as a percentage of respondents who scored each item as 1 or 2 (disagreement) or as 3, 4, or 5 (agreement). A positive consensus is reached if the percentage of agreement is greater than 66%. No consensus is reached, when the sum of the responses for a negative consensus (1 and 2) or a positive consensus (3, 4, and 5) is < 66%.

The aim of this project, which took place in Italy between January 2022 and April 2022, was to reach a consensus on the diagnosis and management of depression in comorbidity with two main neurodegenerative diseases, as Alzheimer’s disease (results published separately) and PD. The survey was developed by a board of nine Italian experts in the two diseases object of the study (7 neurologist, 1 psychiatrist, 1 geriatrician). After reviewing the published literature on the topic, the board met to discuss the main areas of interest and identified four major topics: depression and neurodegenerative diseases, clinical features of depression in patients with Parkinson’s disease, diagnostic criteria of depression in Parkinson’s disease, and treatment of depression in PD. Each topic was subdivided in a variable number of statements corresponding to items where greater need of clarification and debate existed. The survey was then distributed via an online platform to 53 panellists who met the following profile: neurologist or geriatrician, at least 10-year experience in PD, visiting at least 10 patients per week, gender balance (50% women), and equally allocated in the various Italian areas. The vote was anonymous, and no compensation was given to the board or to any of the identified voters. Participants responded to the 30 items of the questionnaire on the first round.

Fifty-three physicians were invited by e-mail to answer the on-line Delphi questionnaire. No personal data were collected from the panel of experts, except for the email addresses that were used only for sending invitation and reminder emails.

## Results

Amongst the 53 panelists selected for the survey, 41 were neurologists and 12 geriatricians; a high response rate was obtained, and 40 specialists responded to the on-line questionnaire 75% (*n* = 53).

A strong consensus was reached for most of the statements in the four topics (25/30 statements, 83.3% of agreement, mean score 75.4), suggesting a shared view of Italian specialists on the selected topics. Panellists acknowledged that depression is strongly associated with neurodegenerative diseases and agreed that depression may precede the onset of motor dysfunctions. Depression was recognised as integral part of the clinical picture of PD, with a severity that is unrelated to motor deficit but can tend to worsen in association with motor fluctuations. The neuropathological correlates of the disease itself were believed to contribute to the genesis of depression in PD and to be at the base of the variability of response to the different therapeutic options adopted in PD. Suicidal behavioural was believed not to be associated to PD whilst the anhedonia was confirmed as one of the prominent aspects of depression in PD.

Tables 1, 2, 3, and 4 summarize the statements and the percentage of agreement/disagreement reached for each one, based on the responses of the 40 panellists.

**Table 1:**
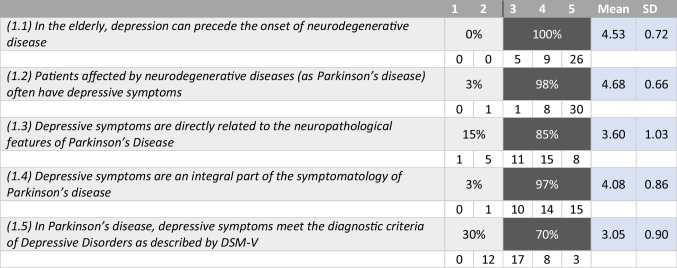
Depression and neurodegenerative disease

**Table 2:**
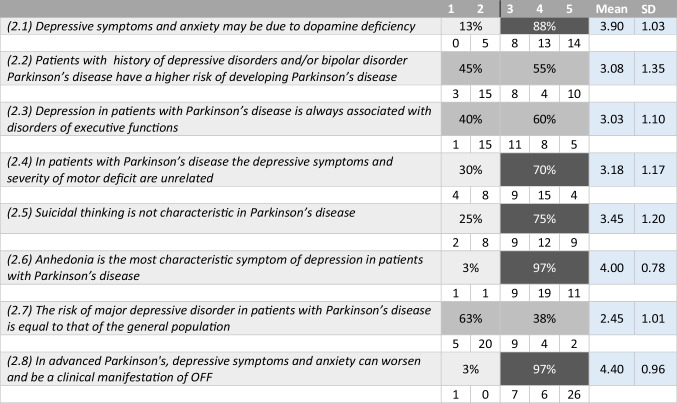
Clinical features of depression in PD

**Table 3:**
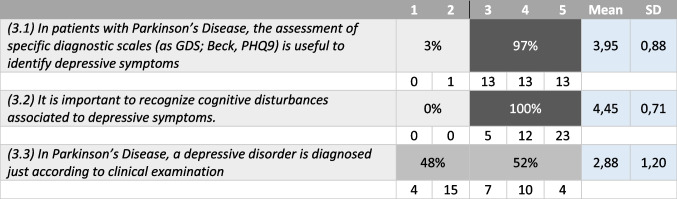
Diagnostic criteria of depression in PD

**Table 4:**
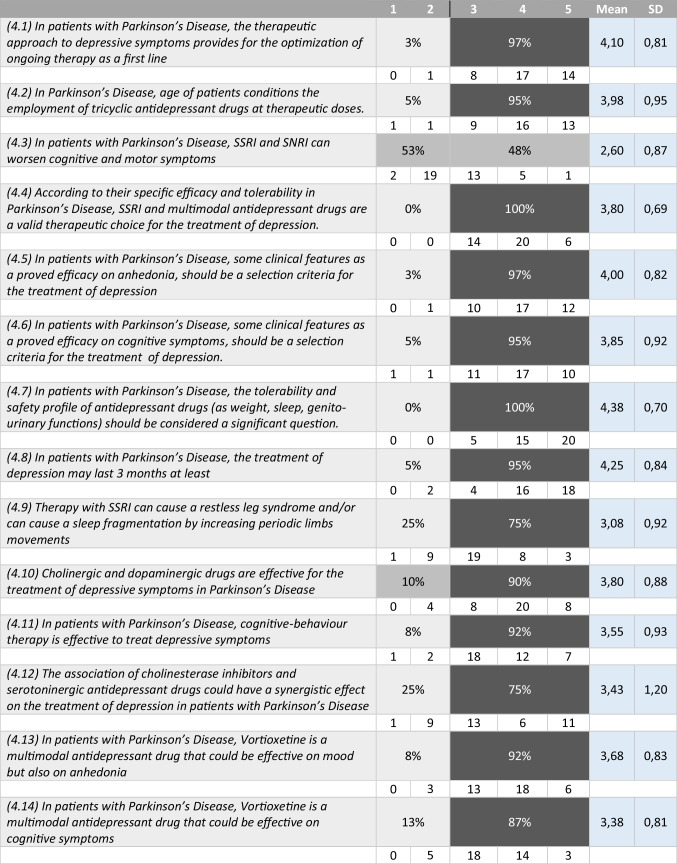
Treatment of depression in patients with Parkinson’s disease

### Topic 1: depression and neurodegenerative disease

#### Statements 1.1–1.5 (Table 1)

An unanimous consensus was reached regarding the statement that depressive symptoms can precede the onset of the neurodegenerative disease (100%) and a very strong consensus was achieved on the two statements that clarify that patients with neurodegenerative diseases often exhibit depressive symptoms (98%) and that these are amongst the core features of PD (97%). The panellists also agreed that the neuropathological abnormalities typical of PD are responsible for the presence of depression before and during the course of the disease (85%). Panellists’ opinions on the statement that the diagnostic criteria for depression according to DSM-V would apply to depression in PD were less convergent, even if a positive consensus was obtained (70%).

### Topic 2: clinical feature of depression in PD

#### Statements 2.1–2.8 (Table 2)

Ninety-seven percent of the panellists agreed with the statement that the anhedonia is one of the prominent features of depression in PD and 75% of them believed that suicidal ideation is not a common trait of this disease. Most of the panellists (88%) believed that depression and anxiety in PD are related to the dopaminergic deficit. As expected, a general agreement supported the statement that, in advanced PD patients, anxiety and depression symptoms can worsen during the off state (97%) and 70% of the experts found that there is no correlation between depression and severity of motor symptoms. No agreement was reached on the following statements: higher risk to develop PD in depressed and bipolar patients (45 versus 55%), correlation between depression and executive dysfunctions (40 versus 60%), and higher risk of PD patients to develop major depression disorders (63 versus 38%).

### Topic 3: diagnostic criteria of depression in PD

#### Statement 3.1–3.3 (Table 3)

There was full agreement on the importance to recognize cognitive disturbance associated to depressive symptoms in PD patients (100%) and that the evaluation scales are useful to identify depressive symptoms in PD patients (97%). No agreement was reached regarding the possible diagnosis of depression based solely on the clinical evaluation (48 versus 53%).

### Topic 4: treatment of depression in patients with PD

#### Statements 4.1–4.14

All panellists agreed that the optimization of the antiparkinsonian therapy is the first step in the therapeutic approach to depression in PD patients (97%). Ninety percent of the interviewed concurred that dopamine agonists could be an effective option in the management of depression in PD. It was recognised that the antidepressant treatment should last not less than 3 months (95%) and that the safety profile of the molecule should be of paramount importance in the choice of the class of medications/compound to use (100%). On this regard, panellists acknowledged that the use of SSRI can be associated to a restless leg syndrome (RLS) and to deterioration of the sleep pattern by increasing periodic limb movements (75%). The same percentage of agreement was achieved for the need of continuous monitoring when this class of drugs is used in association with MAO-B inhibitors due to the possibility of inducing a serotoninergic syndrome (75%). A large consensus was reached on the statement that highlights how the use of tricyclics is conditioned by the age of the patients (95%). Despite these careful considerations, no agreement was reached by the experts on the possibility that the use of SSRI and SNRI can worsen motor symptoms and cognitive dysfunctions in PD patients (53 vs. 48%). SSRI and multimodal antidepressants were unanimously considered an effective therapeutic option for depression in PD (100%). A strong consensus was reached on the statements that the possibility to address the anhedonia (97%) and the cognitive impairment (95%) should be taken into consideration when choosing type of antidepressant. The new multimodal antidepressant, vortioxetine, was believed to be effective on the anhedonia by 92% of the panellists, and effective in the amelioration of cognitive dysfunctions by 87% of the experts. Finally, a large consensus was reached on the benefit of the cognitive behavioural therapy in addressing depression in PD (92%).

## Discussion

The results of this Delphi survey have highlighted a shared view amongst Italian neurologists and geriatricians on the neuropathological bases and the characteristics of depression in PD and have underlined the areas where further debate or clarification is needed.

In agreement with the current knowledge, all the panellists confirmed that the presence of depression can precede the onset of motor symptoms [4] and that depression is a frequent comorbidity in neurodegenerative diseases. It was also recognised that in PD, depression is strictly related to the neuropathological changes typical of the disease. Panellists have also strongly agreed that the dopaminergic deficit is responsible for the presence of depression and anxiety in PD patients. There is a large number of evidence that links depression to abnormalities in the dopaminergic, serotoninergic, and noradrenergic system. Neuroimaging studies in depressed PD subjects have shown alteration of the meso-corticolimbic dopaminergic pathway, with degeneration of projections directed to the ventral striatum, the orbitofrontal cortex, the anterior cingulate cortex, and thalamus [9–11]. Abnormalities are also found in serotonergic areas like the hippocampus, the raphe nuclei, and the temporal and frontal cortex. Areas of noradrenergic transmission like the locus coeruleus are primarily involved in the neurodegenerative process of PD and have also been associated to depression in PD [12, 13].

Regarding the clinical features of depression in PD, anhedonia has been confirmed by the experts as a characteristic trait of depression PD patients. It must be noted that anhedonia can be detected even in non-depressed PD patients and that its genesis can be explained by the degeneration of the mesolimbic areas and the resulting dysfunction of the dopaminergic reward pathway [14]. This non-motor symptom, defined as inability to feel pleasure, seems to affect 6.7–45.7% of patients, with a higher frequency amongst depressed patients [14–16].

Most of the panellists believed that suicidal ideation is not frequent in PD. However, recent evidence suggests that this perception may not be true and that suicide in PD is actually more common than initially thought [17]. A recent systematic review highlighted that suicidal ideation is reported in about 30% of parkinsonian patients, with a higher prevalence amongst younger males’ patients, whilst a recent prospective study found that suicide was about 2 times more frequent in patients with PD than in the general population [18]. Moreover, suicide has been reported in PD patients undergoing invasive therapies such as deep brain stimulation and levodopa jejunal infusion, irrespective of clinical motor outcome [19, 20].

Consensus was not reached regarding the statement that patients with history of major depression and/or bipolar disorder have higher risk to develop PD. These findings reflect the uncertainty in literature; despite scientific evidence emerged in the last 20 years have associated a history of depressive symptoms with an increased risk of PD, it remains to be established whether depression is an independent risk factor or merely a prodromic manifestation of PD [4].

The epidemiological studies performed had different design and used different diagnostic criteria for depression, ranging from questionnaire to physician-based evaluation according to different classification systems (the International Classification of Health Problems in Primary Care, International Classification of Disease, or the Diagnostic and Statistical Manual of Mental Disorders (DSM) criteria). Most of the studies were not able to distinguish between major and minor depression and dysthymia since generic diagnostic criteria were used to select the cases (e.g., ICD-8) [4]. In studies specifically addressing clinical manifestations [1, 21, 22], most of the patients had milder forms of depression, based on DSM criteria. In agreement with this, 70 percent of the experts have confirmed that the spectrum of depressive disorders in PD meets the diagnostic criteria of Depressive Disorder as described by DSM-V (S. 1.5). The diagnosis of minor depression in PD population is based on the presence of milder and fewer symptoms compared to major depression. Moreover, the overlapping between some of PD symptoms (namely psychomotor retardation, insomnia, weight loss, fatigue) and those typical of major depression represents a challenge in characterizing major depression in PD population [23]. It needs to be also considered that the depressive symptoms such as apathy, lack of motivation, and reduced cognitive performance may also overlap with an initial cognitive impairment. In a recent metanalysis, depression and apathy were found to be strongly associated with mild cognitive impairment in PD population [24]. Whilst the presence of MCI is considered a marker to predict the development of dementia [25], the presence of depression is not unanimously considered as a predictive factor for the development of dementia [26]. The opposite results obtained from these studies may be imputable to different study designs and again to the difficulty of weighing the effect of shared symptoms on the cognitive decline.

In regard to the diagnosis of depression, consensus was not reached on the statement that the depression in PD can be diagnosed based solely on the clinical evaluation (S. 3.3 No agreement) and most of the experts agreed that the use of diagnostic scales is helpful in detecting mood abnormalities in parkinsonian patients. This is line with what is reported in literature where most depression rating scales have been found to have good validity and reliability in PD, even though do not clearly distinguish between somatic symptoms due to PD and due to depression [27]. Moreover, the most common approach to evaluate presence and severity of all symptoms independently from their nature, i.e. the “inclusive approach” versus the etiologic approach, has been found to increase reliability of the adopted scales [28]. Amongst clinician-rated scales, the Hamilton Depression Rating Scale is recommended as screening tool as well to assess depression severity. The Geriatric Depression Scale, the Hamilton Depression Inventory, and the Beck Depression Inventory were found to be valid self-reporting instruments to screen, diagnose, and assess depression’s severity in PD patients [27].

When reviewing the therapeutic approach, almost all panellists have agreed that the optimization of the antiparkinsonian treatment is the first approach in controlling depressive symptoms, in particular when the clinical picture is complicated by the presence of motor fluctuations [29]. A general consensus was reached on the statement 2.8 which highlights how depression and anxiety symptoms can be exacerbated or triggered by the OFF state (non-motor off) [30]. Dopamine agonists were confirmed by 90% of the experts as useful adjuvant in the treatment of depression. According to current MDS guidelines the dopamine agonist pramipexole is considered effective in treating depressive symptoms [29]. Nevertheless, special monitoring is required when pramipexole is used in depressed, younger male patients with personality trait characterized by impulsivity, greater search for novelty, and gratification for the risk to trigger an impulse control disorder [31].

Serotoninergic as well as multimodal antidepressants have been recognized as valid therapeutic option for the treatment of depression in PD (100% of agreement) [29, 32], even if SSRI have been linked to the risk of potential worsening of restless leg syndrome and to sleep fragmentation [33]. In line with the inconsistency reported in literature, a consensus was not achieved on the statement that highlights how SSRI and SNRI can induce a worsening of motor symptoms. Some studies have in fact reported the onset of movement disorders following the introduction of these antidepressants [34, 35] whilst trials performed in PD population with both classes have not proved to cause a deterioration of motor performance [32]. Despite what reported in literature, experts have also agreed that the association of SSRI and MAO-B inhibitors requires special monitoring for the risk of causing serotonin syndrome. Studies performed on both reversible and irreversible compounds have demonstrated in fact that the risk of serotonin syndrome in parkinsonian patients treated with MAO-B inhibitors and antidepressant remains extremely low and did not appear to be greater than that encountered in routine medical therapy [36].

Most of the experts have agreed that the use of tricyclics must be guided by the patients’ age and that the safety profile of the compound must necessarily be taken into account when choosing an antidepressant. Cognitive impairment, swallowing abnormalities, urinary dysfunctions, constipation, and orthostatic hypotension are common non-motor symptoms of PD and the use of compounds with anticholinergic activity may exacerbate these problems, especially in older adults or advanced PD patients [29, 32, 37]. Indeed, a randomized study of 3-month sertraline (50 mg) vs. low-dose amitriptyline (25 mg) demonstrated a significant improvement in depressive symptoms with both drugs but only sertraline treatment determined a significant benefit on quality of life (PDQ-39 scale) [38].

Panellists have speculated that vortioxetine, a new multimodal antidepressant labelled for major depressive disorder, may have some potential benefits on anhedonia and cognitive symptoms [39]. A pooled analysis of trials performed in patients with MDD showed significant short-term efficacy against anhedonia [40] and other studies have suggested that vortioxetine has promising effects in improving cognition in adult and older adults with depressive symptom [40]. These pro-cognitive effects of vortioxetine are thought to be related to the ability of this compound to modulate several neurotransmitters and to increase Ach availability [41]. Finally, the panellists believed that the antidepressant treatment, once optimized, should be maintained for not less than 3 months to ensure efficacy.

## Conclusion

The consensus reached in this Delphi study provides an overview of depression in Parkinson’s disease analysing its aetiology, its fundamental clinical features and its relationship with motor dysfunctions, and the validity of current therapeutic options. Experts have acknowledged that depression is an established risk factor of PD which finds its anatomic substrate in the neuropathological abnormalities that precede the onset of motor symptoms and accompany disease progression. For this reason, they have recognised that depression is one of the characteristic symptoms of PD and an early diagnosis and treatment are necessary to avoid long-term complications. SSRI and SNRI have been confirmed as a valid therapeutic option in the treatment of depression in PD, whilst panellists have agreed that the use of tricyclics must be conditioned by patients’ biological age due to the known anticholinergic adverse effects. The need for addressing cognitive abnormalities as well as the anhedonia typical of PD has recalled the attention versus the use of multimodal antidepressants as innovative drugs in the treatment of depression in Parkinsonian patients thanks to their ability to modulate various neurotransmitter systems.

## Data Availability

The data that support the findings of this study are available from the corresponding author upon reasonable request.
